# The Challenge of Urban Heat Exposure under Climate Change: An Analysis of Cities in the Sustainable Healthy Urban Environments (SHUE) Database

**DOI:** 10.3390/cli5040093

**Published:** 2017-12-13

**Authors:** James Milner, Colin Harpham, Jonathon Taylor, Mike Davies, Corinne Le Quéré, Andy Haines, Paul Wilkinson

**Affiliations:** 1 Department of Social & Environmental Health Research, London School of Hygiene & Tropical Medicine, 15-17 Tavistock Place, London WC1H 9SH, UK; 2 Climatic Research Unit, School of Environmental Sciences, University of East Anglia, Norwich Research Park, Norwich NR4 7TJ, UK; 3 UCL Institute for Environmental Design & Engineering, University College London, Central House, 14 Upper Woburn Place, London WC1H 0NN, UK; 4 Tyndall Centre for Climate Change Research, School of Environmental Sciences, University of East Anglia, Norwich Research Park, Norwich NR4 7TJ, UK

**Keywords:** climate change, urban heat, temperature, sustainability, urban health

## Abstract

The so far largely unabated emissions of greenhouse gases (GHGs) are expected to increase global temperatures substantially over this century. We quantify the patterns of increases for 246 globally-representative cities in the Sustainable Healthy Urban Environments (SHUE) database. We used an ensemble of 18 global climate models (GCMs) run under a low (RCP2.6) and high (RCP8.5) emissions scenario to estimate the increase in monthly mean temperatures by 2050 and 2100 based on 30-year averages. Model simulations were from the Coupled Model Inter-comparison Project Phase 5 (CMIP5). Annual mean temperature increases were 0.93 degrees Celsius by 2050 and 1.10 degrees Celsius by 2100 under RCP2.6, and 1.27 and 4.15 degrees Celsius under RCP8.5, but with substantial city-to-city variation. By 2100, under RCP2.6 no city exceeds an increase in T_mean_ > 2 degrees Celsius (relative to a 2017 baseline), while all do under RCP8.5, some with increases in T_mean_ close to, or even greater than, 7 degrees Celsius. The increases were greatest in cities of mid to high latitude, in humid temperate and dry climate regions, and with large seasonal variation in temperature. Cities are likely to experience large increases in hottest month mean temperatures under high GHG emissions trajectories, which will often present substantial challenges to adaptation and health protection.

## 1 Introduction

The world is becoming increasingly urbanized. Cities are already home to more than half of the world’s population [1], they generate around 85% of global GDP and are responsible for up to 76% of energy-related greenhouse gas (GHG) emissions [2]. They are therefore a key focus for actions to help mitigate climate change—actions that have the potential for appreciable ancillary benefits to public health through reduction of harmful exposures (e.g., air pollution) and promotion of healthier behaviours in such areas as diet and physical activity [3].

However, the populations of cities are also potentially vulnerable to the consequences of climate change, including the direct effects of increased heat [4]. Some degree of global warming is inevitable from the GHGs that anthropogenic activity has already contributed to atmospheric concentrations of GHGs [5]. A key question and challenge for society is the extent to which future emissions can be reduced in order to contain global warming to less damaging limits.

For assessing climate change impacts and adaptation responses at fine spatial scales, such as those of regions and cities, dynamical downscaling techniques can be applied to regional climate models (RCMs), driven by global climate models (GCMs) [6]. However, the available ensemble of outputs from downscaling initiatives, such as CORDEX [7], is not yet consistent from region to region. There remains a need for more consistent estimates of future temperatures in order to perform assessments across large numbers of globally-distributed cities.

In this paper, we use a large ensemble of GCMs to examine the patterns of temperature rise that may be expected in cities across the globe under a high and a low GHG emissions trajectory. The analysis is based on data from the Sustainable Health Urban Environments (SHUE) project, which has developed a database of information on a globally-distributed sample of cities [8]. The broad aim of the SHUE project is to support research on the responses to environmental risks to health and the potential impacts for health of strategies for sustainable urban development. The database contains a wide range of information on city characteristics, environmental risks (such as air pollution), and markers of urban form and energy use. This paper describes the climate change data held in the database and demonstrates its application to improving understanding of the benefits of strong climate change mitigation efforts.

## 2 Materials and Methods

The SHUE database includes information on a random sample of 246 global cities with populations over 15,000 obtained from GeoNames [9], stratified by national wealth in terms of Gross National Income (GNI) per capita (<US$1045, US$1045–4125, US$4125–12,746, >US$12,746) [10], population size (<100 K, 100 K–500 K, 500 K–1 M, 1 M–5 M, >5 M), and Bailey’s ecoregion ‘domain’ (dry, humid temperate, humid tropical, polar) [11]. The sample size of 246 was based on calculations of statistical power appropriate for comparative analyses of variables across cities in the database. A further 63 cities were added to this sample, primarily to include cities with specific characteristics and/or policies related to environmental sustainability. Here, we focus only on the 246 randomly-selected cities in the database (Table 1).

### 2.1 Temperature-Related Climate Change Risk

Monthly simulated climate data was estimated for SHUE cities using 18 GCMs under a low GHG emissions scenario, Representative Concentration Pathway (RCP) 2.6, and a high emissions scenario, RCP8.5 [12–15]. RCP2.6 gives a global average temperature change consistent with the 2015 Paris Agreement (i.e., a change in global temperature of less than 2 °C relative to a pre-industrial baseline), while RCP8.5 is broadly representative of business-as-usual.

The model simulations were from Coupled Model Intercomparison Project Phase 5 (CMPI5) [16], which provided major input to the Fifth Assessment Report of the Intergovernmental Panel on Climate Change (IPCC) [17]. Mean monthly temperature data for 1901–2100 was downloaded from the main CMIP5 data repository (via the Earth System Grid Federation (ESGF)–http://pcmdi9.llnl.gov). 18 GCMs were available for this variable (Table 2). Since each model has a different grid resolution, all models were interpolated to a standard 0.5 degrees latitude × 0.5 degrees longitude grid.

A simple bias adjustment was performed at the grid box level for each GCM in order to improve agreement with observations, in this case the CRU-TSv3.22 dataset (which is provided on the same 0.5 degree grid) [18]. In this simple approach, the difference was calculated between the observed and simulated long-term average for 1961–1990. Offset or adjustment factors were calculated for each month and model and then applied in an additive way to the entire simulated series. The assumption underlying any bias adjustment approach is that model biases are stationary.

The final step in pre-processing the GCM data was to take the set of latitude and longitude coordinates for the 246 SHUE cities and to extract the data for the grid box in which each city is located. In coastal areas, the nearest land grid box was used.

### 2.2 City-Level Characteristics

**Figure 1:**
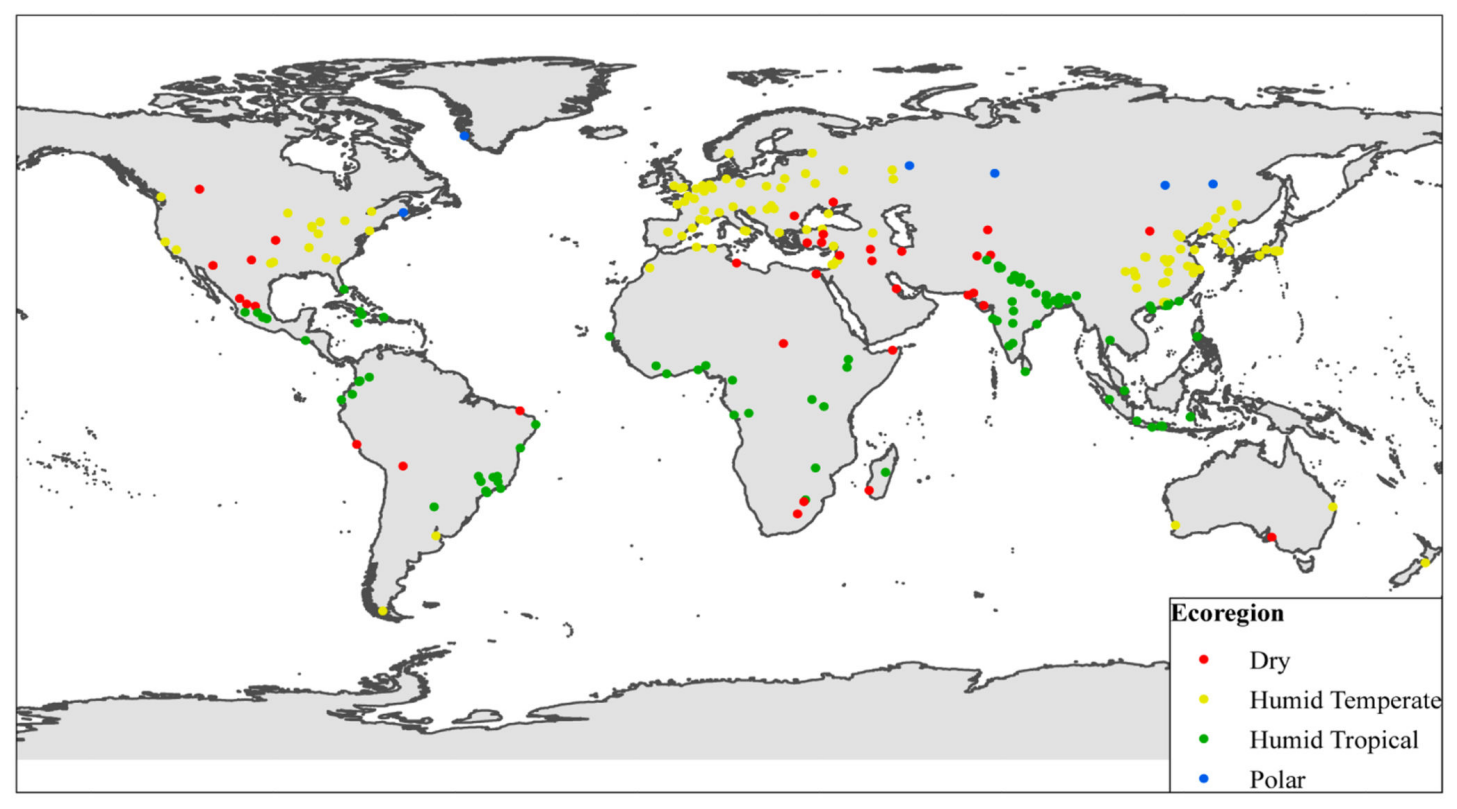
Geographical distribution of SHUE cities and their classification with regard to ecoregion domain.

The climate data were combined with the following information on the characteristics of each city, where available:

- Location: the coordinates (latitude and longitude) of each city obtained from GeoNames [9].
- Population size: estimates of city populations obtained from GeoNames.
- Ecoregion: the Bailey’s ecoregion in which the city is located (Figure 1). The ecoregion is a hierarchical system based on climate, vegetation, geomorphology, and soil characteristics [11]. We used only the upper ‘domain’ level of classification.

### 2.3 Analyses

For each city, we calculated 30-year averages of monthly T_mean_ for a baseline period (1988–2017, referred to as 2017), a near-future period (2021–2050, referred to as 2050), and a far-future period (2071–2100, referred to as 2100). The ensemble mean (mean of all 18 GCMs) was calculated, together with the annual mean for all series (mean of all 12 monthly values). Analyses presented here are based on changes in the annual average of T_mean_ and for the hottest and coldest months of the year in 2050 and 2100 compared with 2017. The model outputs are presented as changes from the baseline period, rather than as absolute values. The focus on changes in temperature (together with the bias adjustment described above) should help to reduce, though not eliminate, the impact on the analysis of model biases and shortcomings, including some of those related to the relatively coarse spatial scale of the GCMs [19].

Changes in temperatures for SHUE cities estimated by the GCMs were analysed in relation to markers of their geographical location, including their coordinates (latitude/longitude), Bailey’s ecoregion domain, and WHO region (Africa, Americas, Eastern Mediterranean, Europe, South-East Asia, Western Pacific). We also analysed the results in relation to city population size. The analyses were performed using simple tabulation and graphical methods, including analyses of both uni-variate and bi-variate distributions. Where there was no data on city-level characteristics for a given city, the city was excluded from that part of the analysis.

## 3 Results

### 3.1 Temperature Changes by 2050 and 2100

Based on the simple average across the 18 GCMs, the mean annual temperature increase in SHUE cities (relative to 2017) was estimated to be 0.93 degrees Celsius by 2050 and 1.10 degrees Celsius by 2100 under RCP2.6, and 1.27 degrees Celsius by 2050 and 4.15 degrees Celsius by 2100 under RCP8.5 (Table 3). The corresponding figures for the hottest month of the year were 1.01 and 1.17 degrees Celsius by 2050 and 2100, respectively, under RCP2.6, and 1.38 and 4.48 degrees Celsius under RCP8.5. This emphasizes the relatively modest increases in temperature by mid-century under each of these GHG emissions pathways, but the much greater changes by the end of the century unless there is a unless there is a rapid reduction of GHG emissions to bring the pathway much closer to that of RCP26.

**Figure 2:**
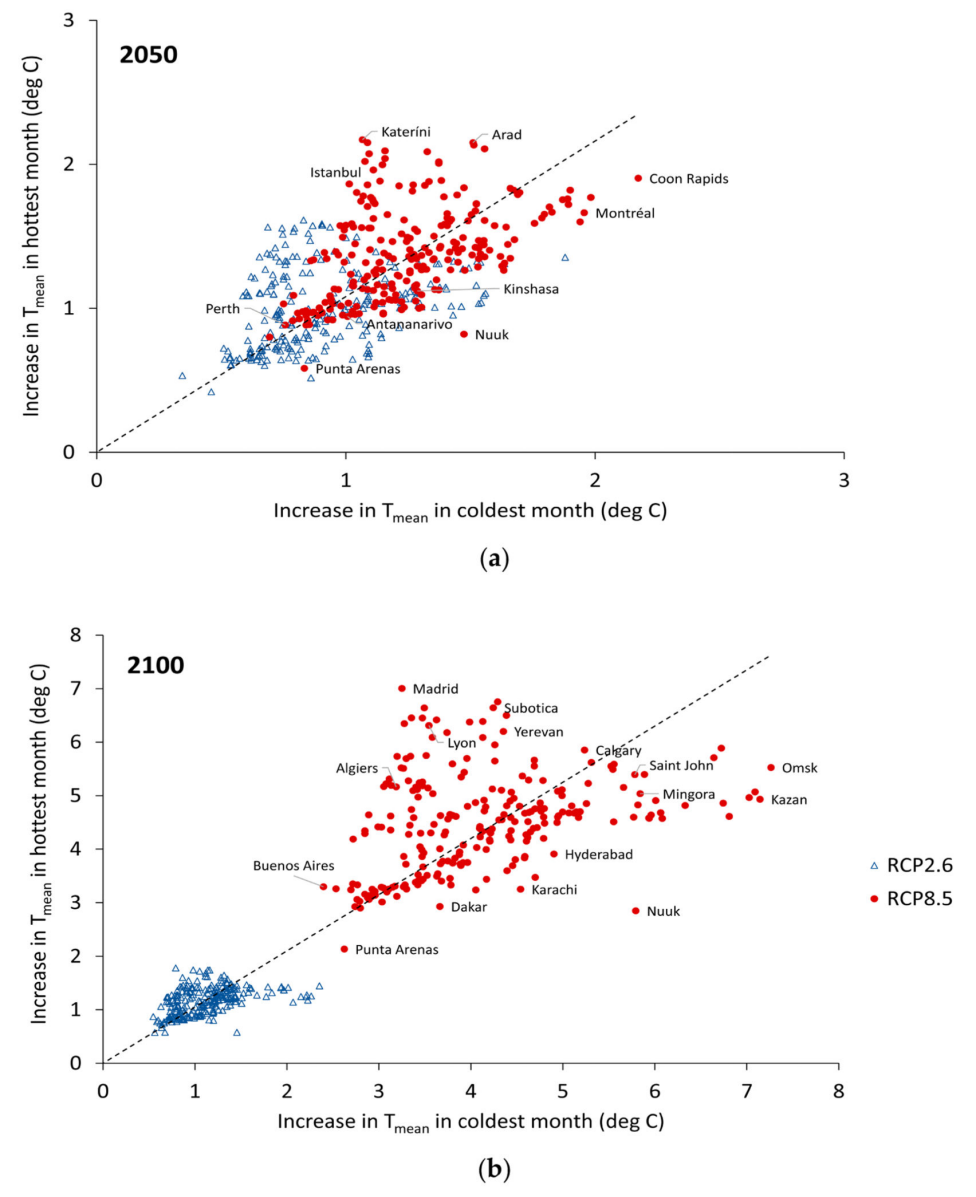
Increase in T_mean_ in the hottest month vs. in the coldest month (retative to 2017) under RCP2.6 (blue open triangles) and RCP8.5 (red dots) by: (**a**) the year 2050 and (**b**) the year 2100.

By 2050, there was considerable overlap in the temperature increases experienced in SHUE cities under RCP2.6 and RCP8.5 (Figure 2). There was however substantial city-to-city variation in the GCM results (see Supplementary Materials). By 2050, only a few cities (Adana, Ankara, Arad, Bucharest, Denizli, Karabük, Katerini, Madrid, Mezotúr, Subotica, Zagreb, and Zapotizhzhya) showed an increase in T_mean_ for the hottast month of the year of greatet than 2 degrees Celsius, all under RCP8.5. However, by 2100, all cities exceeded 2 degrees Celsius increase for the hottest month, with the largest increases close to (and in one case, Madrid, exceeding) 7 degrees Celsius. Under RCP8.5, by 2100, the T_mean_ in the hottest month will on average exceed 40 °C in three cities (Dammam, Baghdad, and Amravati).

Cities with large increases for the hottest month of the year generally had comparably large increases for the coldest month. However, some more northerly cities with colder winter climates (Montreal, Moscow, Nuuk, Omsk, and Saint Petersburg) had relatively larger increases for the coldest month by comparison with the hottest month of the year, while southern European cities, including Madrid and Katerini, had relatively large increases in T_mean_ for the hottest month compared with the increases for the coldest month.

### 3.2 Variations in Temperature Change by Latitude, Ecoregion Domain, and City Size

**Figure 3:**
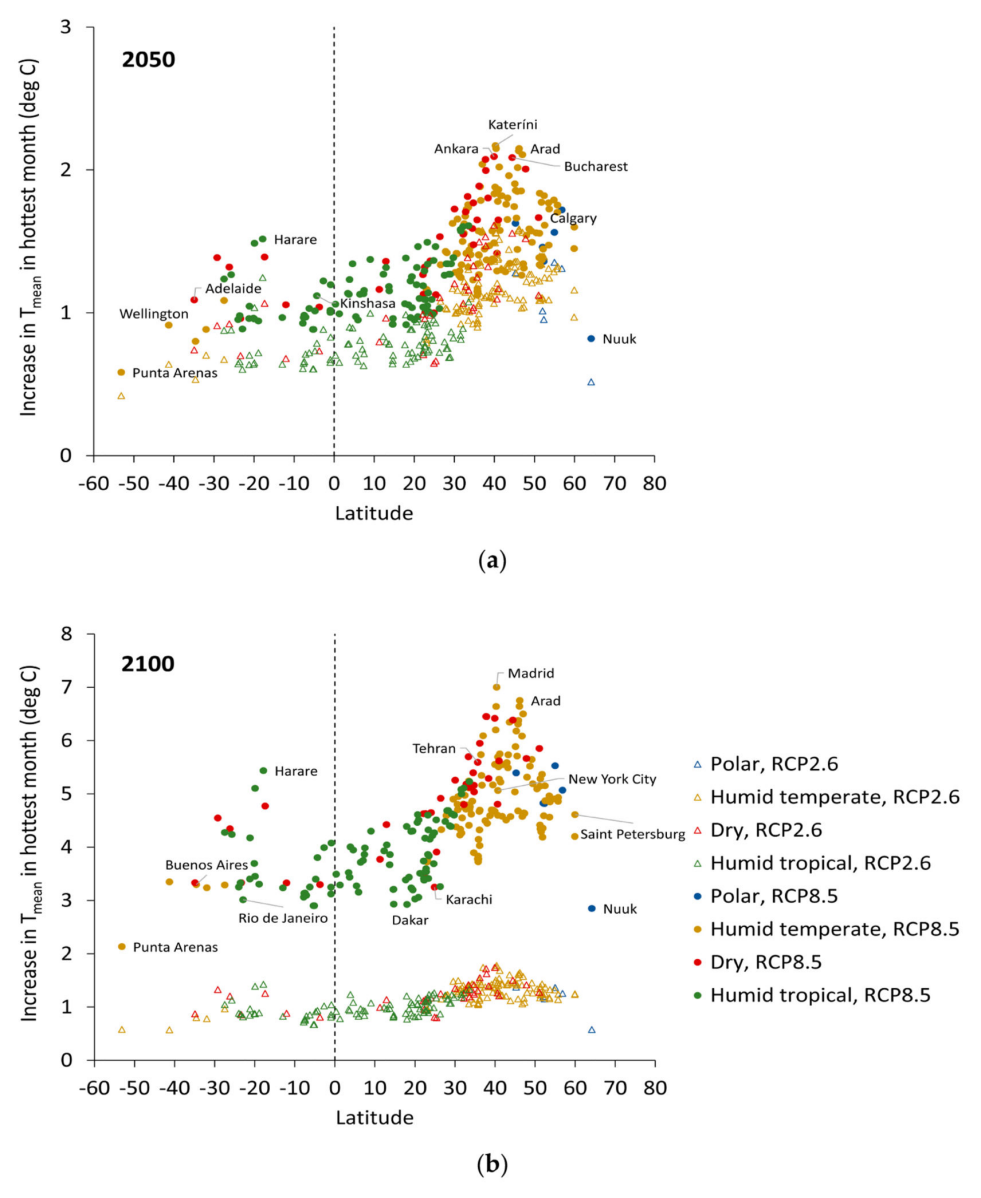
Increase in T_mean_ under RCP2.6 (open triangles) and RCP8.5 (dots) relative to 2017 for the hottest month against latitude by: (**a**) the year 2050 and (**b**) the year 2100. Colouring indicates Bailey’s ecoregion domains.

**Figure 4:**
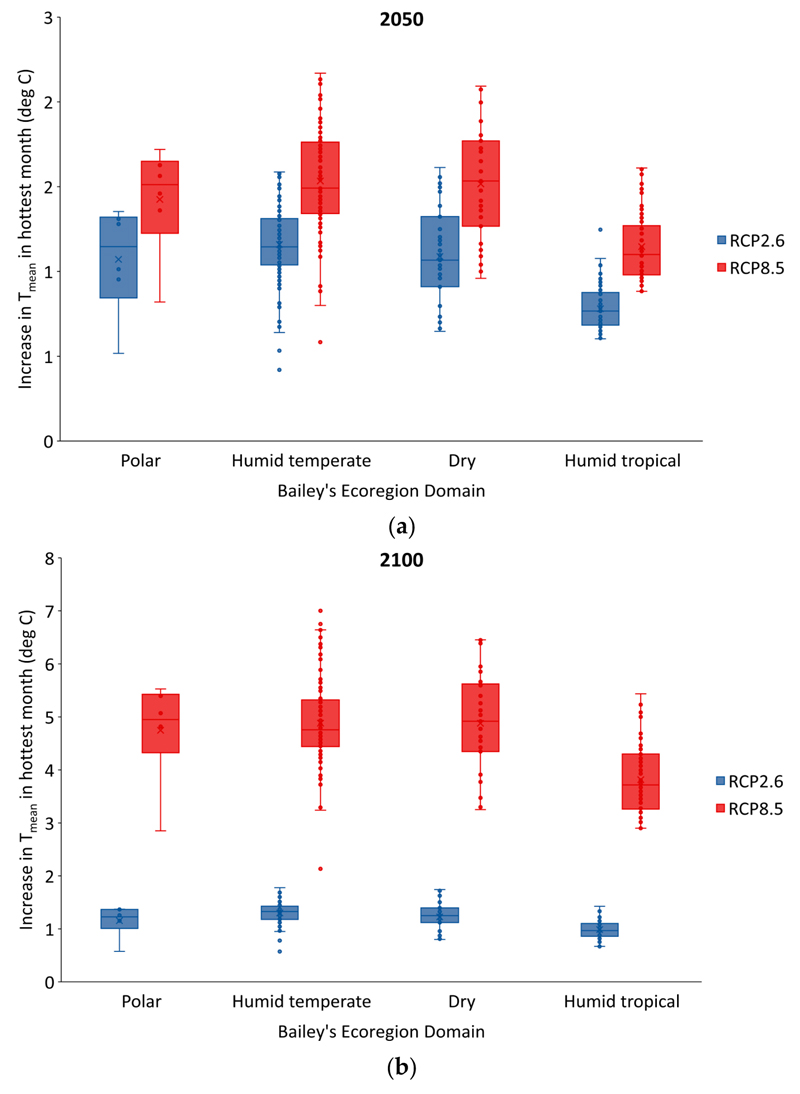
Increases in T_mean_ in the hottest month under RCP2.6 (blue) and RCP8.5 (red) relative to 2017 for cities in different Bailey’s ecoregion domains by: (**a**) the year 2050 and (**b**) the year 2100.

The magnitude of the temperature increases for the hottest month in relation to latitude and ecoregion domain are shown in Figures 3 and 4. Figure 3 shows the well-documented amplification of warming at high-latitudes. The smallest temperature increases are estimated to be those for cities close to the equator, and the largest in cities at latitudes around 40 to 50 degrees north, with somewhat smaller increases at latitudes above this. Corresponding to the latitudinal patterns, the temperature increases for the hottest month were generally largest for (humid) temperate and dry regions, and somewhat lower for humid tropical climates, although there was overlap in the increase across all these categories. Climate differences across ecoregions may affect the ability of cities to adapt to increasing temperatures—for example, the ability to provide greening for urban cooling.

The differences in temperature changes in the hottest and coldest months by ecoregion domain (Figure 4) are potentially important because of differences in diurnal and seasonal temperature variations in each region. Although the increment in T_mean_ for the hottest month was smallest for humid tropical regions, cities in these regions tend to have high relative and absolute humidity, and small diurnal and seasonal variation in ambient temperatures. Cities in the temperate and dry regions, however, with the largest temperature increments for the T_mean_ of the hottest month according to our estimates, tend to have generally lower relative humidity and appreciably greater diurnal and seasonal variation.

**Figure 5:**
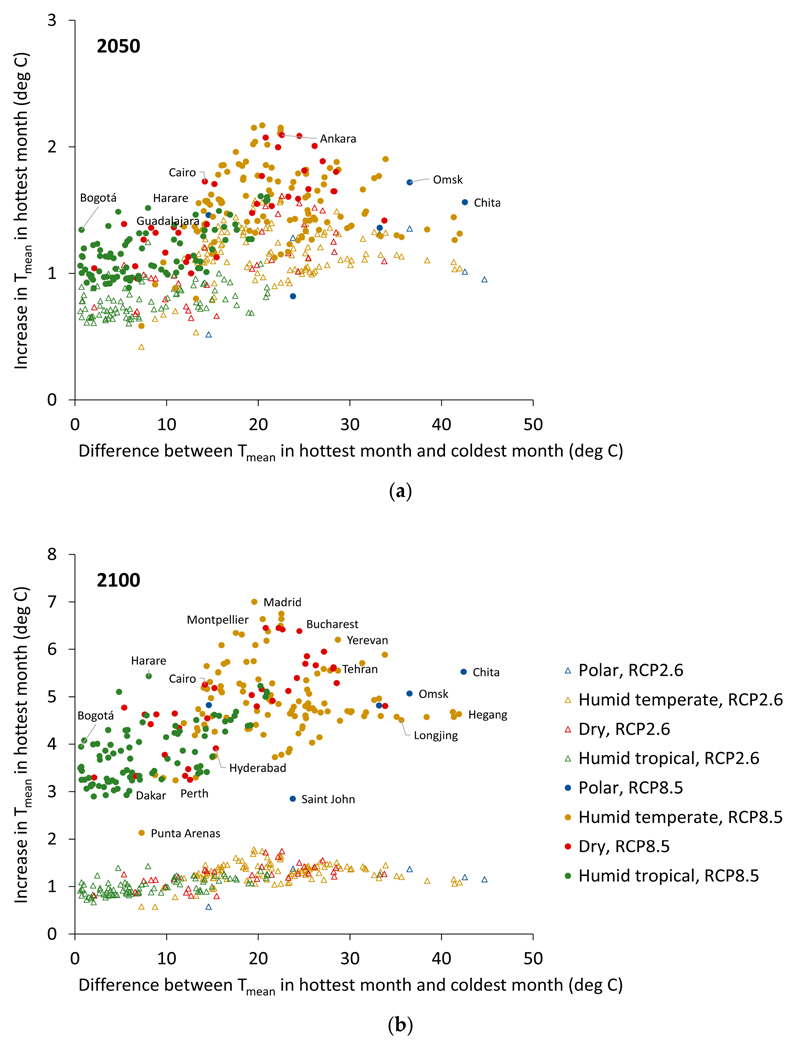
Increases in T_mean_ for the hottest month relative to 2017: relationship to the seasonal variation in temperature as represented by the difference between the T_mean_ for the hottest and coldest month by (**a**) the year 2050 and (**b**) the year 2100. Colouring indicates Bailey’s ecoregion domain.

Figure 5 shows the generally inverse relationship between the increase in T_mean_ and the winter–summer differences in temperature as reflected by the difference in T_mean_ of the hottest and coldest months of the year. The figure demonstrates the considerable challenges for adaptation faced by cities that experience substantial warming during the hottest month of the year yet also extremely cold wintertime conditions.

**Figure 6:**
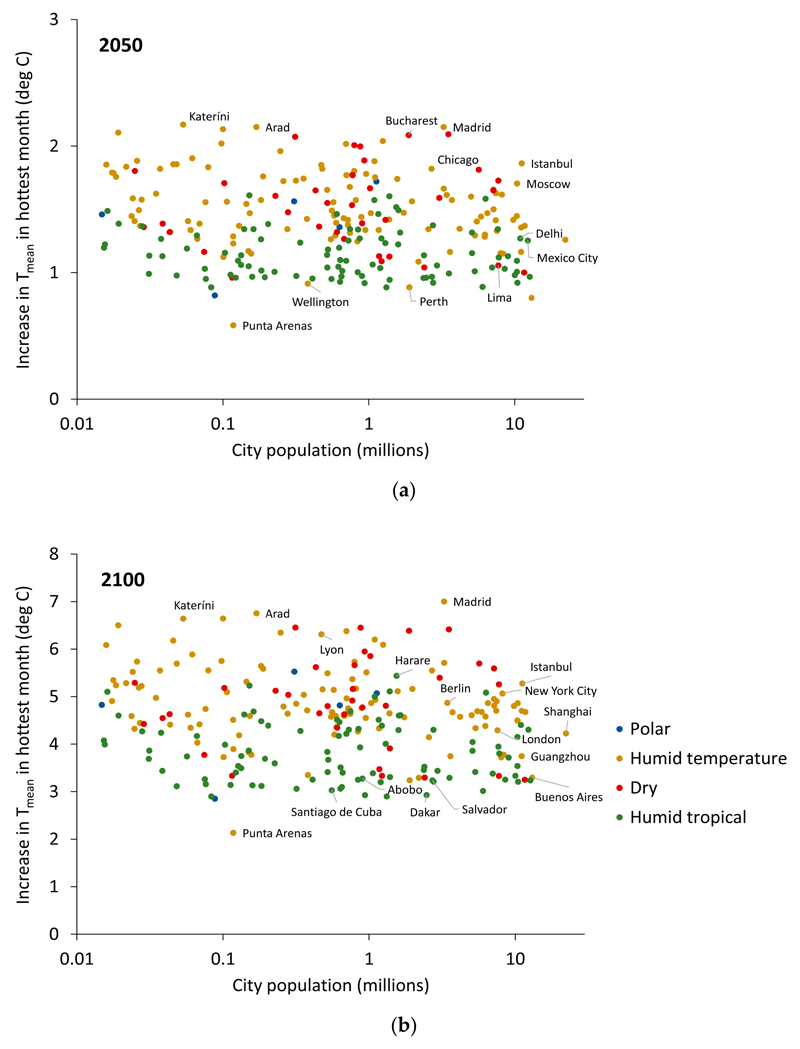
Increase in T_mean_ in the hottest month by 2100 (relative to 2017) under RCP8.5 vs. city population size (log scale) by (**a**) the year 2050 and (**b**) the year 2100. Colouring indicates Bailey’s ecoregion domain.

There was no clear pattern of association between the temperature increase for the hottest month and city size (population) (Figure 6). The temperature increases for SHUE ‘Megacities’ over 10 million in population (*n* = 16) are in the range of 3 degrees Celsius to just over 5 degrees Celsius. It is worth noting that Figure 6 represents only present day populations; these populations are likely to increase considerably over the century, especially in Asia and Africa. The ability of cities to adapt may vary depending on their size, with smaller but growing cities better able to implement the necessary heat adaptation infrastructure, and on their wealth, with richer cities better able to afford mitigation measures.

## 4 Discussion

The results of these analysis provide an insight into the temperature increases that may occur in cities over this century under high and low GHG emissions trajectories. The results confirm substantial increases in hottest month temperatures in all cities under RCP8.5 by the end of the century, with many likely to experience increases of 4 to 7 degrees Celsius, especially in cities at higher latitudes with temperate or dry climates. More generally, the work demonstrates the desirability of having consistent data on a large and globally-representative sample of cities. Such information can enable a better understanding of interactions between climate change and other environmental health issues, including the potential to achieve co-benefits across multiple risks through actions to improve urban sustainability [8].

Although we do not here attempt to quantify the health and social impacts of these temperature rises, in a recent multi-country analysis of temperature-related mortality, the ‘optimum temperature’ (i.e., the point of minimum mortality in relation to daily temperature), was found to vary from around the 60th percentile of the daily distribution in tropical areas to around the 80–90th percentile in temperate regions [20]. Hence, a 4 to 7 degrees Celsius rise in T_mean_ for the hottest month would usually represent an appreciable increase above the threshold for heat deaths [20–23], and often to temperatures that are well beyond the current distribution. Such increases are likely to present considerable challenges to adaptation and health protection, especially for cities where the mean temperature (i.e., calculated using both day and night temperatures) for the whole month rises above core body temperature of 37 degrees Celsius—as it does in several locations in our analysis.

However, the variation in temperature increases with regard to latitude and ecoregion domain may also indicate different implications for adaptation responses. Although cities in low latitude tropical climates generally had more modest increases in T_mean_ for the hottest month, these cities also tend to have high humidity and small diurnal and seasonal variations in temperature. The combination of heat and humidity (as reflected by high wet bulb globe temperatures and similar indices) presents a particular physiological stress [24,25] that may be greater than that generated by higher but drier ambient temperatures. Also, in tropical climates there is usually limited nocturnal relief. In contrast, the cities in temperate and dry climates with larger potential temperature increases have much greater diurnal and seasonal variation in both temperatures and humidity, which offer potential options to help control indoor environments through the buffering effect of thermal mass [26], or to differentiate activities across the year to reduce exposure to the harshest temperatures for outdoor workers [27].

The data we present do not provide information on future changes in extremes of temperature. However, the fact that the temperature increases for the hottest month were generally similar to the temperature increase for the whole year (Table 3) suggests that most of the increase in exposure to the highest temperatures may occur because of the upward shift in the mean temperature distribution rather than by increasing the frequency of temperature extremes by spreading the distribution at a given mean. We acknowledge, however, that these monthly means are not necessarily very sensitive to changes at the tails of the temperature distribution. Moreover, this conclusion of course depends on the ability of current GCMs to capture distributional shifts accurately. In any event, an upward shift in the mean temperature for the year or the hottest month would lead to exposures that are very rare or non-existent under the current climate conditions—and hence by definition present extreme challenges for current heat adaptation strategies.

Among the main strengths of our study is the fact that the analysis was of a globally representative sample of cities and so reflects variation of urban populations with regard to region, city size, and economic development. Its results were also based on an ensemble of 18 global climate models, though here we show only mean values rather than presenting the suite of individual model results to show the diversity of their results. On the other hand, the analysis was of change in (dry bulb) temperatures alone, taking no account humidity, or evidence on the precise form of the distribution of daily temperatures. Moreover, the analyses did not attempt to incorporate the urban heat island (UHI) effect—the name given to the occurrence of higher outdoor temperatures in metropolitan areas compared with those of the surrounding countryside caused by the thermal properties (heat absorption, capacity, conductance, and albedo) of the surfaces and materials found in urban landscapes, the reduced evapotranspiration from reduced natural vegetation, and the waste heat production from anthropogenic activities [28–30]. The UHI effect may add to the more general effect of increasing temperatures, especially in larger cities, though the implications for personal exposure and health are more complex [31,32] and surface temperature effects may in part be offset by overshadowing by high rise buildings in urban centres [33]. A further limitation was the relatively coarse resolution of the GCMs, resulting in an inability to distinguish some cities from neighbouring cities and potential biases for coastal cities. However, our focus on changes in temperatures (rather than absolute values) should have minimized these effects.

Combined assessment of temperature and humidity, UHI effects, and more detailed assessment of the implications for health are all important steps for further research, therefore. However, the main policy implications are clear. The first is the importance of aggressive reduction in GHG emissions in order to help reduce the more extreme temperature rises for urban populations that could be expected from emissions trajectories similar to that represented by RCP8.5. The second is to ensure adaptation planning takes account of the likelihood of very sizeable temperature increases in urban centres around the globe that will lead many populations to be exposed to temperatures well beyond those of their current experience.

## Supplementary Material

The following are available online at www.mdpi.com/2225-1154/5/4/93/s1.

Table S1 and S2

## Figures and Tables

**Table 1:** List of SHUE cities by WHO region and Bailey’s ecoregion domain.

WHO Region 1	Ecoregion Domain	Cities
Africa	Polar	(none)
	Humid temperate	Algiers, Didouche Mourad
	Dry	Benoni, Thaba Nchu, Toliara
	Humid tropical	Abobo, Addis Ababa, Antananarivo, Dakar, Ekangala, Harare, Hawassa, Ikerre, Kinshasa, Lagos, Ntungamo, Pointe-Noire, Usagara, Vavoua, Yaoundé
Americas	Polar	Saint John
	Humid temperate	Alpharetta, Augusta, Benicia, Buenos Aires, Calumet City, Carmel, Chicago, Coon Rapids, Corcoran, Fort Worth, Grand Rapids, Hamilton, Montréal, Murray, New York City, Plano, Punta Arenas, Richmond
	Dry	Calgary, Cochabamba, Emporia, Fortaleza, Jerez de García Salinas, Lima, Lubbock, San Luis Potosí, Tucson, Victoria de Durango
	Humid tropical	Álvaro Obregón, Barbacena, Belo Horizonte, Bogotá, Cali, Conceição das Alagoas, Corrientes, Deerfield Beach, Divinópolis, El Cerrito, Guadalajara, Holguín, Ibarra, João Pessoa, Kingston, Manta, Mexico City, Puebla, Ribeirão Preto, Rio de Janeiro, Salvador, San Salvador, Santiago de Cuba, Santiago de los Caballeros, Santiago de Querétaro, Santos, São Paulo
Eastern Mediterranean	Polar	(none)
	Humid temperate	Damascus, Marrakesh, Qatana
	Dry	Baghdad, Bosaso, Cairo, Dammam, Erbil, Homs, Hyderabad, Kabul, Karachi, Mingora, Sabratah, Tehran, Zalingei
	Humid tropical	Gujranwala, Kohat, Lahore
Europe	Polar	Chita, Izhevsk, Nuuk, Omsk
	Humid temperate	Adana, Arad, Berlin, Bressanone, Brunoy, Cava Dè Tirreni, Düsseldorf, Farnborough, Gloucester, Gomel, Hadera, Hamburg, Hrodna, Istanbul, Karabük, Kateríni, Kazan, Leczna, Le Grand-Quevilly, Le Mans, Lódz, London, Lyepyel, Lyon, Madrid, Marseille, Mezotúr, Montpellier, Moscow, Namur, Nantes, Napoli, Oostend, Oslo, Rotterdam, Saint Petersburg, Sant Vicenç dels Horts, Simferopol, Subotica, Tolyatti, Valencia, Vercelli, Voorst, Yerevan, Zagreb
	Dry	Ankara, Bucharest, Denizli, Konya, Namangan, Zaporizhzhya
	Humid tropical	(none)
South-East Asia	Polar	(none)
	Humid temperate	Hamhung, Songnim
	Dry	Rajkot
	Humid tropical	Amravati, Amritsar, Bahraich, Bangalore, Bangkok, Bareilly, Bhopal, Bidar, Budaun, Buduran, Chaibasa, Delhi, Dhaka, Durgapur, Galesong, Hailakandi, Haldwani, Hisua, Jakarta, Laksar, Makassar, Matara, Meerut, Mojokerto, Mumbai, Mysore, Padang, Pasuruan, Pune, Rajshahi, Ranchi, Shantipur, Shrirampur, Varanasi, Visakhapatnam, Yogyakarta
Western Pacific	Polar	Tahe
	Humid temperate	Beijing, Brisbane, Changchun, Changzhou, Chengdu, Chongqing, Daegu, Dongguan, Foshan, Guangzhou, Guankou, Guiyang, Hangzhou, Harbin, Hegang, Ikoma, Jiamusi, Langfang, Longjing, Nagareyama, Nanchong, Nanjing, Narita, Ome, Perth, Pingdingshan, Qingdao, Seoul, Shanghai, Shenyang, Suzhou, Tai’an, Takayama, Tianjin, Tokyo, Wellington, Wuhan, Xi’an, Xiangtan, Xianyang, Yingkou, Zhoukou, Zhumadian,
	Dry	Adelaide, Baotou
	Humid tropical	Danshui, Hong Kong, Macau, Manila, Shantou, Shenzhen, Singapore, Quezon City, Yashan, Zhanjiang

1 Janin (Palestine) not formally included in any WHO Region (though likely to be Eastern Mediterranean).

**Table 2:** Grid resolution of 18 global climate models used in analysis.

Global Climate Model Acronym	Original Model Resolution (Number of Latitude × Longitude Cells)
CCSM4	192 × 288
CNRM-CM5	128 × 256
CSIRO-Mk3-6-0	96 × 192
CanESM2	64 × 128
GFDL-CM3	90 × 144
GFDL-ESM2G	90 × 144
HadGEM2-ES	145 × 192
IPSL-CM5A-LR	96 × 96
IPSL-CM5A-MR	143 × 144
MIROC-ESM	64 × 128
MIROC-ESM-CHEM	64 × 128
MIROC5	128 × 256
MPI-ESM-LR	96 × 192
MPI-ESM-MR	96 × 192
MRI-CGCM3	160 × 320
NorESM1-M	96 × 144
bcc-csm1-1	64 × 128
bcc-csm1-1-m	160 × 320

**Table 3:** Average of GCM estimates of changes in T_mean_ by 2050 and 2100 (relative to 2017).

WHO Region	Ecoregion Domain	Cities	2050						2100					

			RCP2.6			RCP8.5			RCP2.6			RCP8.5		
			
			Mean (°C)	Coldest Month (°C)	Hottest Month (°C)	Mean (°C)	Coldest Month (°C)	Hottest Month (°C)	Mean (°C)	Coldest Month (°C)	Hottest Month (°C)	Mean (°C)	Coldest Month (°C)	Hottest Month (°C)
Africa	Polar	0	-	-	-	-	-	-	-	-	-	-	-	-
	Humid temperate	2	0.95	0.70	1.24	1.32	1.08	1.72	1.09	0.82	1.36	4.23	3.27	5.45
	Dry	3	0.87	0.86	0.84	1.26	1.27	1.22	1.04	0.97	1.13	4.23	4.01	4.08
	Humid tropical	15	0.80	0.79	0.82	1.15	1.14	1.16	0.94	0.89	1.00	3.79	3.74	3.87

Americas	Polar	1	1.26	1.49	1.28	1.59	1.79	1.63	1.50	1.96	1.37	5.03	5.78	5.39
	Humid temperate	18	1.05	1.04	1.14	1.42	1.48	1.54	1.25	1.39	1.26	4.44	4.41	4.90
	Dry	10	0.97	0.91	1.01	1.35	1.33	1.41	1.12	1.17	1.14	4.36	4.11	4.64
	Humid tropical	27	0.77	0.74	0.76	1.10	1.02	1.09	0.92	0.87	0.94	3.61	3.41	3.65

Eastern Mediterranean	Polar	0	-	-	-	-	-	-	-	-	-	-	-	-
	Humid temperate	3	0.97	0.75	1.18	1.35	1.16	1.58	1.12	0.92	1.33	4.30	3.52	4.87
	Dry	13	0.99	0.98	1.08	1.37	1.30	1.52	1.17	1.15	1.22	4.54	4.27	4.89
	Humid tropical	3	0.94	1.13	0.94	1.49	1.46	1.60	1.24	1.28	1.28	5.06	5.07	5.14

Europe	Polar	4	1.25	1.46	1.05	1.64	1.61	1.39	1.41	1.76	1.10	5.37	6.49	4.57
	Humid temperate	45	0.99	0.85	1.31	1.34	1.24	1.72	1.16	1.14	1.38	4.20	3.99	5.35
	Dry	6	1.08	0.91	1.48	1.48	1.27	1.98	1.27	1.27	1.54	4.75	4.10	6.17
	Humid tropical	0	-	-	-	-	-	-	-	-	-	-	-	-

South-East Asia	Polar	0	-	-	-	-	-	-	-	-	-	-	-	-
	Humid temperate	2	1.13	1.28	1.13	1.44	1.50	1.48	1.28	1.40	1.38	4.64	5.15	4.63
	Dry	1	0.77	1.09	0.71	1.15	1.36	1.13	0.95	1.20	0.96	3.83	4.70	3.47
	Humid tropical	36	0.73	0.82	0.78	1.10	1.16	1.18	0.95	1.03	1.02	3.81	4.04	3.91

Western Pacific	Polar	1	1.23	1.43	0.95	1.65	1.62	1.36	1.36	1.44	1.15	5.39	6.33	4.82
	Humid temperate	43	1.05	1.13	1.01	1.35	1.38	1.32	1.22	1.26	1.22	4.38	4.53	4.36
	Dry	2	0.94	0.94	0.92	1.25	1.16	1.25	1.04	0.96	1.07	4.04	3.93	4.07
	Humid tropical	10	0.76	0.74	0.73	1.05	1.10	1.04	0.97	1.03	0.89	3.41	3.38	3.42
