# Supplementary material for: The Challenge of Urban Heat Exposure under Climate Change: An Analysis of Cities in the Sustainable Healthy Urban Environments (SHUE) Database
Source: Climate (Basel). Author manuscript; Available in PMC 2019 Jul 8. (PMC6614032; doi:10.3390/cli5040093)
Supplement: Table S1 and S2 [file EMS83178-supplement-Table_S1_and_S2.pdf]

Supplementary materials

The Challenge of Urban Heat Exposure under Climate Change: An Analysis of Cities in the Sustainable Healthy Urban Environments (SHUE) Database

This appendix accompanies the paper "The Challenge of Urban Heat Exposure under Climate Change: An Analysis of Cities in the Sustainable Healthy Urban Environments (SHUE) Database". It provides two additional tables not presented in the main paper showing the inter-city range of the estimated temperature increases.

Table S1. Minimum of GCM estimates of changes in T_{mean} by 2050 and 2100 (relative to 2017).

WHO Region	Ecoregion Domain	Cities	2050						2100					
			RCP2.6			RCP8.5			RCP2.6			RCP8.5		
			Mean (°C)	Coldest month (°C)	Hottest month (°C)	Mean (°C)	Coldest month (°C)	Hottest month (°C)	Mean (°C)	Coldest month (°C)	Hottest month (°C)	Mean (°C)	Coldest month (°C)	Hottest month (°C)
Africa	Polar	0	-	-	-	-	-	-	-	-	-	-	-	-
	Humid temperate	2	0.92	0.68	1.12	1.27	1.03	1.56	1.07	0.80	1.28	4.08	3.19	5.16
	Dry	3	0.69	0.62	0.70	1.01	1.02	0.96	0.81	0.73	0.86	3.40	3.28	3.33
	Humid tropical	15	0.67	0.65	0.63	0.99	0.90	0.94	0.81	0.74	0.84	3.21	3.05	2.93
Americas	Polar	1	1.26	1.49	1.28	1.59	1.79	1.63	1.50	1.96	1.37	5.03	5.78	5.39
	Humid temperate	18	0.41	0.34	0.42	0.69	0.69	0.58	0.54	0.60	0.58	2.22	2.40	2.13
	Dry	10	0.69	0.68	0.68	0.96	0.92	1.04	0.81	0.73	0.81	3.22	3.27	3.30
	Humid tropical	27	0.64	0.51	0.60	0.88	0.75	0.89	0.77	0.55	0.77	2.85	2.53	2.93
Eastern Mediterranean	Polar	0	-	-	-	-	-	-	-	-	-	-	-	-
	Humid temperate	3	0.93	0.73	0.92	1.31	1.11	1.25	1.07	0.81	1.18	4.25	3.42	4.27
	Dry	13	0.77	0.72	0.65	1.12	1.09	1.00	0.96	0.91	0.80	3.63	3.40	3.25
	Humid tropical	3	0.88	1.08	0.84	1.47	1.40	1.58	1.19	1.23	1.24	4.99	4.94	5.08
Europe	Polar	4	0.79	0.86	0.52	1.21	1.43	0.82	1.04	1.35	0.58	4.35	5.79	2.85
	Humid temperate	45	0.78	0.59	0.97	1.02	0.86	1.33	0.87	0.69	1.14	3.21	2.72	4.19
	Dry	6	1.03	0.79	1.17	1.42	1.09	1.65	1.22	1.05	1.21	4.53	3.35	5.62
	Humid tropical	0	-	-	-	-	-	-	-	-	-	-	-	-
South-East Asia	Polar	0	-	-	-	-	-	-	-	-	-	-	-	-
	Humid temperate	2	1.12	1.27	1.11	1.44	1.47	1.47	1.27	1.36	1.37	4.60	5.12	4.59
	Dry	1	0.77	1.09	0.71	1.15	1.36	1.13	0.95	1.20	0.96	3.83	4.70	3.47
	Humid tropical	36	0.60	0.51	0.61	0.87	0.81	0.88	0.69	0.62	0.67	2.89	2.80	2.90
Western Pacific	Polar	1	1.23	1.43	0.95	1.65	1.62	1.36	1.36	1.44	1.15	5.39	6.33	4.82
	Humid temperate	43	0.58	0.53	0.64	0.81	0.76	0.88	0.56	0.56	0.57	2.88	2.70	3.24
	Dry	2	0.67	0.66	0.74	0.95	0.79	1.09	0.78	0.72	0.87	3.10	2.77	3.33
	Humid tropical	10	0.60	0.61	0.64	0.86	0.93	0.92	0.75	0.80	0.78	2.96	2.94	3.21

Table S2. Maximum of GCM estimates of changes in T_{mean} by 2050 and 2100 (relative to 2017).

WHO Region	Ecoregion Domain	Cities	2050						2100					
			RCP2.6			RCP8.5			RCP2.6			RCP8.5		
			Mean (°C)	Coldest month (°C)	Hottest month (°C)	Mean (°C)	Coldest month (°C)	Hottest month (°C)	Mean (°C)	Coldest month (°C)	Hottest month (°C)	Mean (°C)	Coldest month (°C)	Hottest month (°C)
Africa	Polar	0	-	-	-	-	-	-	-	-	-	-	-	-
	Humid temperate	2	0.99	0.72	1.36	1.37	1.14	1.88	1.12	0.84	1.43	4.37	3.34	5.74
	Dry	3	0.97	1.05	0.92	1.39	1.45	1.39	1.17	1.15	1.33	4.66	4.44	4.55
	Humid tropical	15	0.94	1.01	1.25	1.36	1.42	1.52	1.11	1.20	1.43	4.53	4.48	5.44
Americas	Polar	1	1.26	1.49	1.28	1.59	1.79	1.63	1.50	1.96	1.37	5.03	5.78	5.39
	Humid temperate	18	1.38	1.54	1.36	1.86	2.17	1.90	1.65	2.35	1.47	5.60	6.72	5.89
	Dry	10	1.16	1.18	1.32	1.60	1.83	1.80	1.56	2.22	1.40	5.07	5.24	5.85
	Humid tropical	27	0.93	0.97	1.04	1.29	1.26	1.49	1.15	1.15	1.39	4.41	4.33	5.10
Eastern Mediterranean	Polar	0	-	-	-	-	-	-	-	-	-	-	-	-
	Humid temperate	3	1.00	0.76	1.33	1.37	1.26	1.76	1.15	0.98	1.41	4.36	3.71	5.24
	Dry	13	1.18	1.42	1.50	1.65	1.76	1.89	1.42	1.67	1.55	5.52	5.89	5.95
	Humid tropical	3	1.03	1.22	1.08	1.52	1.56	1.61	1.31	1.38	1.33	5.17	5.28	5.23
Europe	Polar	4	1.55	1.88	1.35	1.88	1.89	1.72	1.67	2.26	1.37	6.14	7.26	5.53
	Humid temperate	45	1.42	1.64	1.59	1.85	1.98	2.17	1.65	2.23	1.78	5.60	7.14	7.00
	Dry	6	1.23	1.34	1.61	1.58	1.50	2.09	1.34	1.59	1.74	5.26	5.31	6.45
	Humid tropical	0	-	-	-	-	-	-	-	-	-	-	-	-
South-East Asia	Polar	0	-	-	-	-	-	-	-	-	-	-	-	-
	Humid temperate	2	1.13	1.29	1.15	1.45	1.53	1.49	1.29	1.44	1.39	4.68	5.17	4.67
	Dry	1	0.77	1.09	0.71	1.15	1.36	1.13	0.95	1.20	0.96	3.83	4.70	3.47
	Humid tropical	36	0.94	1.21	1.00	1.46	1.53	1.57	1.18	1.38	1.27	4.95	4.99	5.00
Western Pacific	Polar	1	1.23	1.43	0.95	1.65	1.62	1.36	1.36	1.44	1.15	5.39	6.33	4.82
	Humid temperate	43	1.24	1.56	1.21	1.59	1.66	1.66	1.39	1.53	1.51	5.24	6.08	4.95
	Dry	2	1.20	1.22	1.10	1.55	1.53	1.42	1.30	1.19	1.26	4.97	5.10	4.81
	Humid tropical	10	0.88	0.81	0.80	1.20	1.24	1.19	1.16	1.20	1.00	3.86	3.80	3.74
